# Live birth and maternity outcome in childhood and adolescent cancer survivors under 18 years at diagnosis: a 40-year population-based cohort study

**DOI:** 10.1038/s41416-024-02818-0

**Published:** 2024-09-12

**Authors:** W. H. Wallace, T. W. Kelsey, D. Morrison, R. A. Anderson

**Affiliations:** 1 Department of Paediatric Haematology and Oncology, Royal Hospital for Children and Young People and University of Edinburgh, Edinburgh, UK; 2 School of Computer Science, University of St. Andrews, North Haugh, St. Andrews, UK; 3 Scottish Cancer Registry, Public Health Scotland, 1 South Gyle Crescent, Edinburgh, UK; 4 Centre for Reproductive Health, Institute for Regeneration and Repair, University of Edinburgh, Edinburgh, UK

**Keywords:** Risk factors, Paediatric cancer

## Abstract

**Background:**

Survival from childhood and adolescent cancer has increased, but the chance of a livebirth in female survivors under 18 years at diagnosis may be reduced.

**Methods:**

We performed a national population-based analysis, including all female cancer survivors diagnosed in Scotland before the age of 18 years between 1981 and 2012. Scottish Cancer Registry records were linked to Scottish maternity records. Females from the exposed group with no pregnancies before cancer diagnosis (*n* = 2118) were compared with three general population controls matched for age and year of diagnosis.

**Findings:**

The cumulative incidence of a livebirth for all diagnoses was reduced to 37% (95% CI 33–40%) for cancer survivors at 30 years of age vs 58% (57–60%) for controls. The deficit varying by diagnosis: for lymphoid leukaemia, the cumulative incidence at 30 years was 29% (23–36%) vs 57% (52–61%) for controls with similar deficits in CNS tumours and retinoblastoma. There was a steady improvement in the chance of livebirth in those diagnosed more recently.

**Interpretation:**

We have shown a reduced chance of livebirth in female survivors of cancer diagnosed before age 18. The deficit is present for all diagnoses.

## Introduction

With continued advances in the treatment of young people with cancer, survival has improved such that 5-year survival for all childhood cancers in Europe in 2010–2014 was 81% (95% CI 81–82) [1]. For adolescents and young adults (AYA) (15–24 years), survival has improved over time to 82% [81.1–83.3] in 2005–2007) [2]. The potential to have children after successful treatment for cancer remains a priority for many young women [3–5]. Recent population-based studies in women less than 40 years of age at first diagnosis have demonstrated a significant reduction (~38%) in the chance of a life birth across all cancer diagnoses compared to age-matched controls [6]. In addition, in those who were able to conceive following treatment, family size was smaller (2.0 + /− 0.8 vs 2.3 + /− 1.1 livebirths) [7]. Less is known about the chance of a livebirth in children and young females less than 18 years of age at first diagnosis, for whom fertility may be an important priority.

The North American Childhood Cancer Survivor Study (CCSS) has for many years provided information about reproductive function and pregnancy outcome in a selected cohort of 5-year survivors of the most common types of childhood cancer who were diagnosed before age 21 years and treated at 27 institutions in the USA and Canada between 1970 and 1999 [8–10]. In a subset of the CCSS cohort who had received only chemotherapy or radiotherapy to sites distant to reproductive function [11], female survivors had a decreased chance of having a livebirth compared to sibling controls (female survivors: 0.82, 0.76–0.89; *P* < 0.0001). Other studies have included young adults as well as girls and adolescents and shown comparable evidence of loss of fertility associated with specific treatments [12–14]. Population-based data, without the possibility of selection bias, on childbirth in female survivors of specifically childhood cancer is therefore lacking. In Scotland, the availability of linkable databases of cancer registrations and pregnancy-related outcome records offers the opportunity to study whether girls and young women diagnosed with cancer before aged 18 years achieve pregnancy after a cancer diagnosis on a population basis. We have also studied birthweight and gestational age in this cohort of young cancer survivors and compared them to age and deprivation-matched population-selected controls.

## Methods

Scottish Cancer Registry data is linked to hospital admissions data and death registration data and is in this study linked to the Maternity Inpatient and Day Case dataset (SMR02), which collects episode-level data for every obstetric event together with geographical measures such as postcode-based estimates of household deprivation. In this study Scottish cancer registry, death records and maternity discharge records were linked to form two cohorts for retrospective comparison. The exposed group (*n* = 2118) consists of all female patients aged 0–17 years at first cancer diagnosis in the years 1981 to 2012 with no pregnancy events recorded before the diagnosis. The control group (*n* = 6354) were randomly selected from the Scottish population, three per exposed case, and matched by age at and year of cancer diagnosis, no previous pregnancy and postcode-based index of deprivation that incorporates social class, unemployment, car ownership and overcrowding at the household level [15]. Maternity and death records were linked for all subjects from 1981 to 2018, which was the latest year for which records were available when the data access request was made. Analyses were performed for all diagnoses, and for Surveillance, Epidemiology, and End Results Program (SEER) site groups based on the histologic types and primary site groups given in the International Classification of Childhood Cancer, Third Edition (ICCC-3) (Table 1). Any site group having fewer than ten diagnoses in the period 1981–2012 was omitted from Table 1 but was included in the calculations for all diagnoses.

**Table 1:** (a) Cancer site groups, numbers of patients and controls, ages at diagnosis and combined years of follow-up.

(a)				
Diagnosis	SEER site group	*N*	Age (years)	Follow-up (years)
All cancers	All	E: 2118 C: 6355	8 (3–14)	E: 33,658 C: 101,055
Lymphoid leukaemias	Ia	E: 491 C: 1,473	4 (2.5–9.5)	E: 8577 C: 25,731
Acute myeloid leukaemias	Ib	E: 103 C: 309	7 (1–14)	E: 1150 C: 3450
Chronic myeloproliferative diseases	Ic	E: 14 C: 42	13 (9.8–16)	E: 159 C: 478
Hodgkin lymphomas	IIa	E: 143 C: 429	15 (13–17)	E: 2699 C: 8098
Non-Hodgkin lymphomas	IIb	E: 41 C: 123	14 (11–15)	E: 617 C: 1852
Unspecified lymphomas	IIe	E: 17 C: 51	15 (11–16)	E: 208 C: 624
Ependymomas and choroid plexus tumour	IIIa	E: 29 C: 87	2 (1–7)	E: 227 C: 680
Astrocytomas	IIIb	E: 292 C: 876	8 (4–12)	E: 4185 C: 12,556
Intracranial and intraspinal embryonal tumours	IIIc	E: 87 C: 261	7 (3–9)	E: 937 C: 2810
Other gliomas	IIId	E: 66 C: 198	8 (5–12)	E: 713 C: 2138
Neuroblastoma and ganglioneuroblastoma	IVa	E: 102 C: 306	1 (0–3)	E: 1367 C: 4100
Retinoblastoma	V	E: 55 C: 165	1 (0–2)	E: 1291 C: 3874
Nephroblastoma and other nonepithelial renal tumours	VIa	E: 87 C: 261	3 (1–4)	E: 1580 C: 4739
Hepatoblastoma	VIIa	E: 12 C: 36	1 (0–2)	E: 148 C: 443

(b)				
Diagnosis	SEER site group	*N*	Age (years)	Follow-up (years)
Osteosarcomas	VIIIa	E: 48 C: 144	14 (10–16)	E: 609 C: 1827
Ewing tumour and related sarcomas of bone	VIIIc	E: 59 C: 177	12 (9–14)	E: 679 C: 2037
Other specified malignant bone tumours	VIIId	E: 16 C: 48	13 (9.8–16)	E: 159 C: 478
Unspecified malignant bone tumours	VIIIe	E: 38 C: 114	11 (3.3–15)	E: 507 C: 1521
Rhabdomyosarcomas	IXa	E: 72 C: 216	5 (3–9)	E: 1010 C: 3029
Fibrosarcomas, peripheral nerve sheath tumours, and other fibrous neoplasms	IXb	E: 16 C: 48	14 (11.8–16)	E: 256 C: 767
Intracranial and intraspinal germ cell tumours	Xa	E: 15 C: 45	11 (7.5–12)	E: 271 C: 814
Malignant extracranial and extragonadal germ cell tumours	Xb	E: 17 C: 51	1(0–3)	E: 214 C: 643
Malignant gonadal germ cell tumours	Xc	E: 43 C: 129	14 (11.5–16)	E: 852 C: 2557
Gonadal carcinomas	Xd	E: 13 C: 39	16 (15–17)	E: 248 C: 744
Other and unspecified malignant gonadal tumours	Xe	E: 14 C: 42	13 (0.25–15)	E: 112 C: 337
Thyroid carcinomas	XIb	E: 49 C: 147	15 (14–17)	E: 979 C: 2937
Malignant melanomas	XId	E: 73 C: 219	15 (13–16)	E: 1520 C: 4559
Skin carcinomas	XIe	E: 26 C: 78	16 (15–16)	E: 590 C: 1771
Other and unspecified carcinomas	XIf	E: 74 C: 222	15 (14–16)	E: 1217 C: 3650

All diagnoses and SEER site groups I through VII; (b) cancer site groups, numbers of patients and controls, ages at diagnosis and combined years of follow-up. SEER site groups VIII through XI.

The primary outcome is the first livebirth after the cancer diagnosis, compared using cumulative incidence functions with controls adjusted for the competing risk of death of their match by censoring at the date of death. Controls entered the study on the date of diagnosis for their exposed match and left the study either on the date of the first subsequent livebirth or on the date of death of their exposed match. The date of diagnosis is defined as the date of first consultation at, or admission to, a hospital for that cancer. This is used as the anniversary date for computation of follow-up and survival and as the date of onset for measuring incidence. Gray’s test for equality of cumulative incidence functions was used to estimate overall *P* values for differences in two cumulative incidence curves adjusted for the competing risk of death before the first livebirth. The Kalbfleisch–Prentice method was used for 95% confidence intervals for incidence curves. Maternal age at first livebirth, birthweight, and gestation for all livebirths were compared using the Wilcoxon signed-rank test. Cumulative incidences are compared at chronological ages, as exposed and control cases were matched by age. To assess changes over era of diagnosis, the cohorts were split into three classes with diagnosis in 1980–1990, 1991–2001 or 2002–2012. Relative risks of a subsequent livebirth were calculated for each epoch of diagnosis by dividing into four mutually exclusive cases: exposed with and without subsequent livebirth, and controls with and without subsequent livebirth. Relative risks are compared for exposed and controls with the date of diagnosis in the same period specified by two dates. Confidence intervals for the relative risk statistic were calculated using normal approximation (Wald’s test).

## Results

We identified 2118 females (age at diagnosis: median 8 (interquartile range 3–14 years) with a cancer diagnosis before their 18th birthday in Scotland and compared them to a control group of 6354 age-matched females. There are 33,658 years of follow-up for the exposed group and 101,055 for the controls (Table 1a, b). The commonest diagnosis is lymphoid leukaemia with 491 cases (age at diagnosis: 4 (2.5–9.5) years) and 1473 matched controls with 8577 years of follow-up for the exposed group (Table 1a). Other diagnoses by SEER site group are detailed in Table 1a, b.

**Fig. 1:**
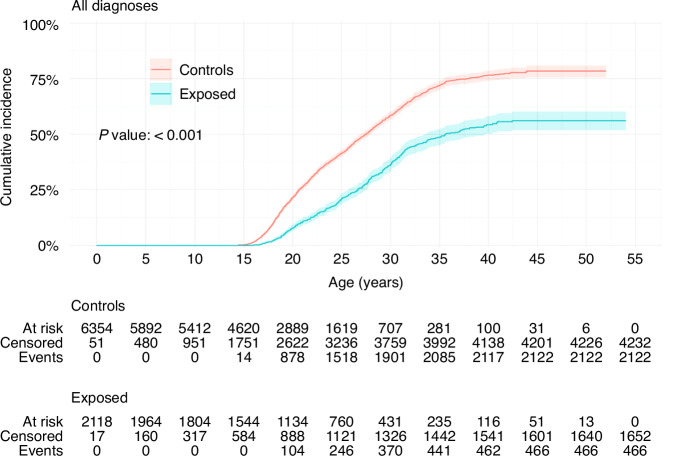
Cumulative incidence of first livebirths in female cancer survivors and matched controls: all diagnoses. Overall *P* value is from Gray’s test for equality of cumulative incidence functions, with all data censored at an age of at most 45 years. Kalbfleisch–Prentice is used for 95% confidence intervals for incidence curves. Events are first livebirths. Censoring is at an age at the end of follow-up or the age of death for an exposed subject, in which case controls are also censored to account for the competing risk of death for their exposed match.

Comparison of all females with a cancer diagnosis under the age of 18 years with their age-matched controls shows that there is a highly significant difference in the chance of a first livebirth (*P* < 0.001) (Fig. 1). At 20 years of age, the cumulative incidence of a first livebirth was 7.9% (6.5–9.4%) for cancer survivors and 22% (20–23%) for matched controls; at 30 years of age the cumulative incidence of a first livebirth was 37% (33–40%) for cancer survivors and 58% (57–60) for matched controls; at 40 years of age the cumulative incidence of a first livebirth was 54% (50–58%) for cancer survivors and 77% (74–79) for matched controls (Table 2a).

**Table 2:** (a) Cumulative incidence of first livebirth at 20, 30 and 40 years of age.

(a)			
SEER site group	20 years	30 years	40 years
All diagnoses			
Controls	22% (20%, 23%)	58% (57%, 60%)	77% (74%, 79%)
Exposed	7.9% (6.5%, 9.4%)	37% (33%, 40%)	54% (50%, 58%)
Acute myeloid leukaemias			
Controls	21% (14%, 28%)	66% (54%, 75%)	−% (−%, −%)
Exposed	12% (4.2%, 24%)	31% (16%, 47%)	42% (22%, 60%)
Lymphoid leukaemias			
Controls	22% (19%, 24%)	57% (52%, 61%)	76% (70%, 81%)
Exposed	5.0% (2.9%, 7.9%)	29% (23%, 36%)	47% (36%, 57%)
Hodgkin lymphomas			
Controls	21% (17%, 26%)	59% (53%, 64%)	78% (71%, 83%)
Exposed	6.1% (2.8%, 11%)	52% (41%, 61%)	72% (60%, 81%)
Non-Hodgkin lymphomas			
Controls	18% (11%, 26%)	51% (37%, 62%)	59% (41%, 73%)
Exposed	9.9% (2.4%, 24%)	19% (6.5%, 36%)	34% (13%, 57%)
Astrocytomas			
Controls	22% (19%, 26%)	58% (53%, 63%)	76% (69%, 82%)
Exposed	6.7% (3.6%, 11%)	33% (24%, 41%)	47% (36%, 57%)
Intracranial and intraspinal embryonal tumours			
Controls	26% (17%, 35%)	64% (49%, 76%)	81% (60%, 92%)
Exposed	3.8% (0.26%, 17%)	12% (2.9%, 29%)	20% (5.3%, 40%)

(b)			
SEER site group	20 years	30 years	40 years
Neuroblastoma			
Controls	16% (10%, 24%)	66% (50%, 78%)	−% (−%, −%)
Exposed	11% (3.4%, 24%)	41% (18%, 63%)	−% (−%, −%)
Retinoblastoma			
Controls	19% (13%, 27%)	61% (48%, 71%)	−% (−%, −%)
Exposed	5.0% (0.88%, 15%)	16% (5.3%, 31%)	−% (−%, −%)
Nephroblastoma			
Controls	23% (17%, 30%)	63% (50%, 74%)	72% (57%, 82%)
Exposed	13% (5.0%, 24%)	32% (16%, 49%)	66% (32%, 86%)
Ewing tumour			
Controls	21% (14%, 30%)	52% (39%, 63%)	60% (46%, 72%)
Exposed	6.3% (11.1%, 18%)	53% (27%, 74%)	53% (27%, 74%)
Rhabdosarcomas			
Controls	18% (11%, 25%)	59% (44%, 71%)	72% (54%, 83%)
Exposed	3.1% (0.22%, 14%)	38% (15%, 62%)	60% (22%, 84%)
Malignant melanomas			
Controls	21% (16%, 27%)	55% (48%, 62%)	79% (69%, 86%)
Exposed	13% (6.3%, 22%)	35% (23%, 47%)	63% (46%, 76%)

All diagnoses and SEER site groups I through VII. Kalbfleisch–Prentice 95% confidence intervals are given; horizontal bars denote insufficient data to derive outputs at that age.

(b) Cumulative incidence of first livebirth at 20, 30 and 40 years of age. SEER site groups VIII through XI. Kalbfleisch–Prentice 95% confidence intervals are given; horizontal bars denote insufficient data to derive outputs at that age.

**Fig. 2:**
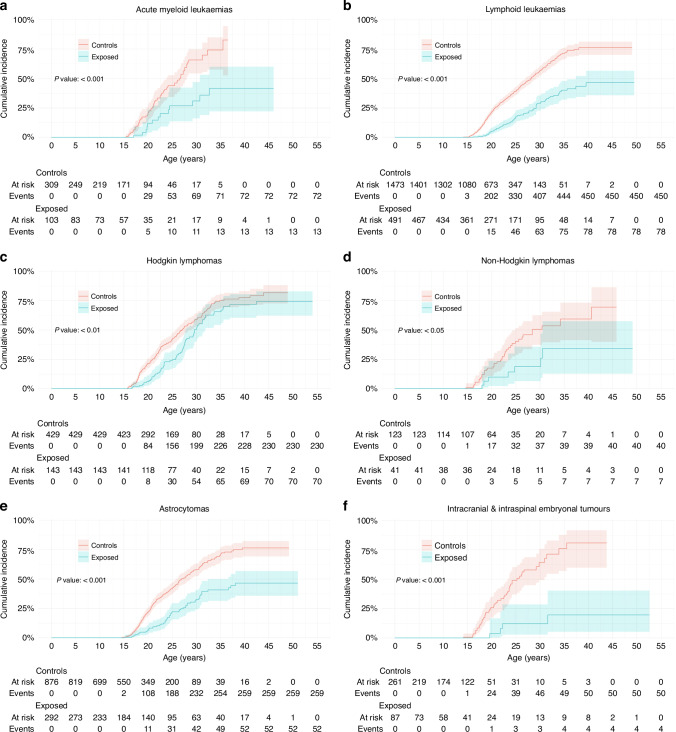

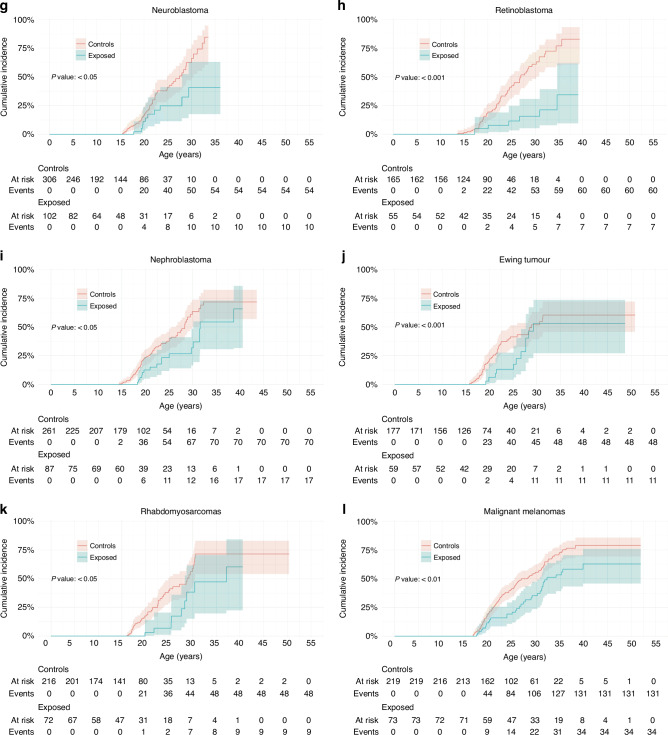
Cumulative incidence of first livebirths in female cancer survivors and matched controls: SEER site groups. **a**–**l** Diagnosis is stratified using histologic types and primary site groups based on the *International Classification of Childhood Cancer, Third Edition*. The overall *P* value is from Gray’s test for equality of cumulative incidence functions, with all data censored at age at most 45 years. Kalbfleisch–Prentice is used for 95% confidence intervals for incidence curves. Events are first livebirth. Controls are adjusted for the competing risk of death for their exposed match.

This deficit was examined for the most common specific diagnoses. For lymphoid leukaemia (the commonest cancer in females age <18 years), the cumulative incidence of a livebirth at age 30 was 29% (23–36%) for survivors and 57% (52–61%) for matched controls (Fig. 2b and Table 2a). However, the deficit was less pronounced for Hodgkin lymphoma where the cumulative incidence of a livebirth at age 30 was 52% (41–61%) for survivors and 59% (53–64%) for matched controls (Fig. 2c and Table 2a). For astrocytoma the difference between exposed and controls at age 30 was both large and highly significant, 14.4% (10.8–18.7%, *n* = 250) for survivors and 31.5% (28.2–34.9%, *n* = 504, *P* < 0.001) for controls (Fig. 2e). Furthermore, except for Ewing tumour (Fig. 2j), the overall cumulative incidence of a livebirth was significantly different from controls for all cancer types studied including sarcoma (rhabdomyosarcoma *P* < 0.05 (Fig. 2k), osteosarcoma [data not shown]), Wilms tumour (nephroblastoma) *P* < 0.05 (Fig. 2i), neuroblastoma *P* < 0.05 (Fig. 2g), retinoblastoma *P* < 0.001 (Fig. 2h), non-Hodgkin lymphoma *P* < 0.05 and malignant melanoma *P* < 0.01 (Fig. 2l).

**Fig. 3:**
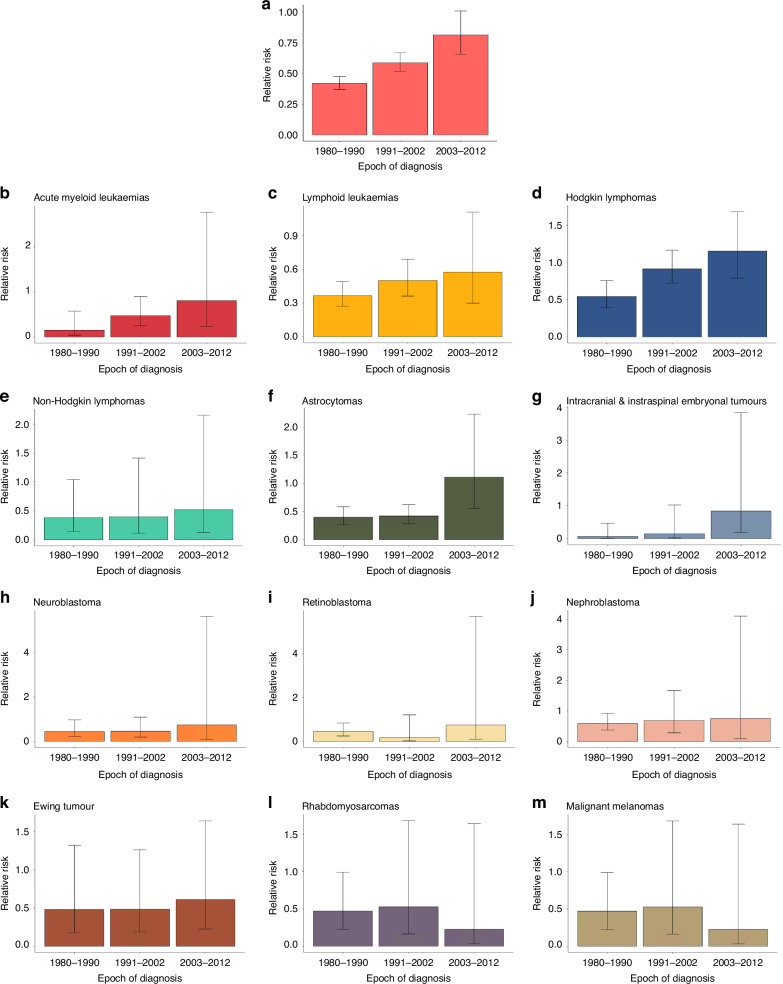
Relative risks of subsequent livebirth for female cancer survivors and matched controls stratified by epoch of diagnosis. **a** Relative risks of subsequent livebirth for female cancer survivors and matched controls stratified by epoch of diagnosis: all diagnoses. A relative risk less than one indicates that a subsequent livebirth is less likely after a cancer diagnosis. Fisher’s exact test is used to calculate 95% confidence intervals. **b**–**m** Relative risks of subsequent livebirth for female cancer survivors and matched controls stratified by epoch of diagnosis: SEER site groups. Diagnosis is stratified using histologic types and primary site groups based on the *International Classification of Childhood Cancer, Third Edition*. A relative risk less than one indicates that a subsequent livebirth is less likely after a cancer diagnosis. Fisher’s exact test is used to calculate 95% confidence intervals.

When analysed by epoch of diagnosis, there was an overall increase in the relative risk of subsequent livebirth from 0.42 (95% CI 0.37–0.48) for 1980–1990 to 0.59 (95% CI 0.52–0.67) for 1991–2002, and to 0.82 (95% CI 0.67–1.01) for 2003–2012 (1980–1990 to 1991–2002 *P* < 0.01; 1991–2002 to 2003–2012 *P* = 0.133; 1980–1990 to 2003–2012 *P* < 0.0001) (Fig. 3a) such that in the most recent epoch the confidence intervals crossed unity. When analysed by diagnosis, there was a steady but not significant improvement in the relative risk of a livebirth for most but not all diagnoses (Fig. 3b–m).

**Fig. 4:**
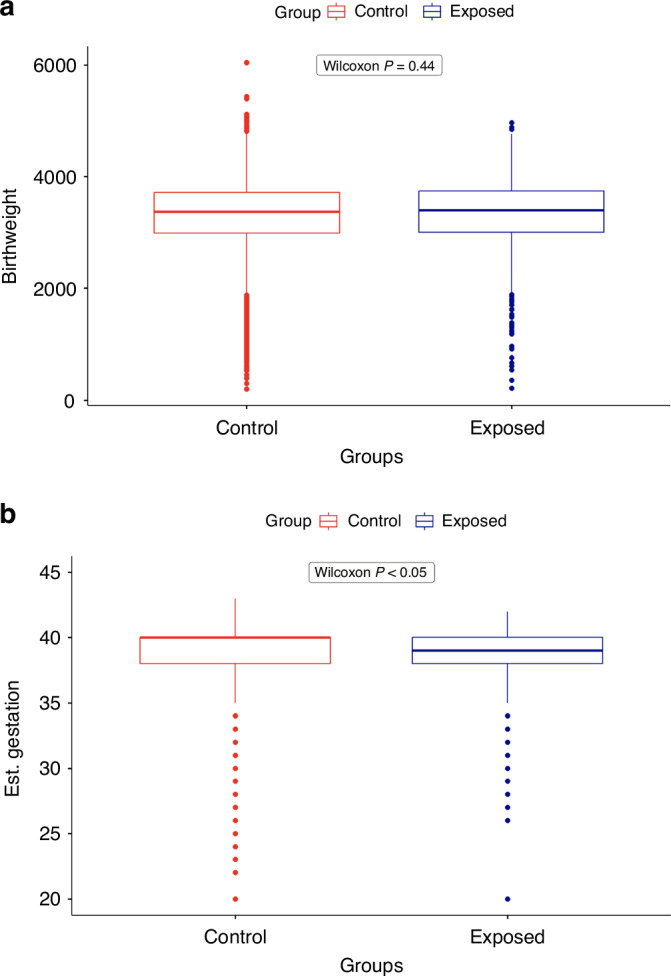
Maternal experience in female cancer survivors and matched controls. No significant differences in median birthweight (**a**). A small but significant difference in median estimated gestation at livebirth (**b**).

Analysis of maternal outcomes revealed there was no significant difference in median birthweight of offspring between survivors (3370 g, IQR 2990–3720 g, *n* = 837) and controls (3400 g, IQR 3000–3740 g, *n* = 6134, *P* = 0.44). There was a small but significant difference in median estimated gestation at livebirth (38.8 weeks IQR 38.4–40.1 weeks in the exposed group versus 39.9 weeks IQR 38.5–40.6 weeks for controls, *P* < 0.05) (Fig. 4), but this did not translate to a significant increase in the prevalence of births before 37 weeks’ gestation (12.4% for exposed versus 13.7% for controls, two-sample z-test for equality of proportions *P* = 0.273).

## Discussion

In this Scottish population study, we have shown that female survivors of cancer diagnosed before their 18^th^ birthday are less likely to have a livebirth when compared to age and deprivation-matched controls. This finding applies across all diagnoses and is most marked in survivors of leukaemia and CNS tumours. It is present even in cancers where exposure to gonadotoxic treatment is minimal, e.g., malignant melanoma where the standard treatment is primarily surgical excision. This analysis provides an holistic overview of the prevalence of childbirth after childhood cancer providing population-based reference data, rather than attempting to attribute possible causes of this, such as direct reproductive toxicity associated with treatment [16], reduced opportunity for parenthood through social difference [17, 18] or potential variations in the desire to have children compared to the general population [5, 19]. With more recent treatment cohorts there was an increased chance of a first livebirth compared to those diagnosed in the 1980s, although even with this long-term national cohort population, group size reduced the power to explore this for specific diagnostic groups and there may well be specific diagnoses where there has not been an improvement. For those survivors who did experience a first livebirth their gestational age was slightly reduced, but birthweight was comparable (Fig. 4).

To our knowledge, this is the first linkage study using a population-based design specifically in childhood cancer. Previous analyses of parenthood after cancer in childhood have generally also included older age groups, preventing specific interpretation of the effect of childhood cancer and its treatment. With improved survival for young people with cancer, the selection of those at high risk of infertility is important as there are now increasingly established opportunities for fertility preservation available [20–23]. In a Finnish population-based study of males and females aged 0–34 years at cancer diagnosis and compared to their siblings, both female and male cancer survivors were approximately 50% less likely to parent at least one child (relative risk (RR) 0.46, 95% CI 0.44–0.48 and RR 0.57, 95% CI 0.54–0.60, respectively). The relative probability of parenthood was especially low in male childhood cancer survivors and female young adult cancer survivors [24]. In contrast, a population-based cohort study of female survivors under age 21 years at diagnosis from Ontario, Canada, showed a relatively small deficit in the cumulative incidence of achieving a pregnancy by age 30 years, 22.3% (95% CI 20.7–23.9%) among survivors vs 26.6% (95% CI 125.6–27.3%) among controls (hazard ratio 0.80, 95% CI 0.75–0.86) [25]. The present analysis which links livebirths to cancer patients aged under 18 years at diagnosis shows results comparable to the Finnish data, with the cumulative incidence of a livebirth at age 30 being 37% for cancer survivors and 58% for matched controls (Fig. 2a). In our previous studies [6, 7] of all women diagnosed with cancer before the age of 40 years in Scotland between 1981 and 2012, we showed that cancer survivors were less likely to achieve a pregnancy when compared to all other Scottish women: Standardised Incidence Ratio (SIR) 0.62 (95% CI: 0.60, 0.63) [6] with the reduced SIR observed for all cancer types. In our recent study, the mean age at first livebirth was greater in cancer survivors compared to age-matched controls overall (31.2 + /− 5.5 vs. 29.7 + /− 6.1 years) [7], and this was confirmed across most diagnoses indicating a cancer-related delay in age at childbirth. In that study, we found that family size (total number of livebirths achieved) was consistently different from controls, being lower overall (2.0 + /− 0.8 livebirths vs. 2.3 + /− 1.1 in controls, *P* < 0.001) with that deficit also present in those diagnosed in childhood. The present data specifically focus on the youngest cancer survivors, as treatment protocols differ from those in adults, and their care is in different, sometimes separate institutions, highlighting the need for specific information for this patient group and their clinical teams. A study from the CCSS [8] of survivors of cancer diagnosed up to age 21 which used siblings as controls with data collection by self-reported questionnaire showed that the RR of a survivor ever being pregnant was 0.81 compared with the sibling cohort. A more recent analysis from the CCSS of chemotherapy exposure (excluding patients treated with radiotherapy that might have affected reproductive function) [11] reported that female survivors chance of having a livebirth was reduced (female survivors: 0.82, 0.76–0.89; *P* < 0.0001) and they concluded that their findings should provide reassurance to most female survivors treated with chemotherapy without radiotherapy to the pelvis or brain. Barton et al. [26] also using data from the CCSS questionnaire-based study including five-year cancer survivors who were less than 21 years old at the time of diagnosis showed survivors had an increased risk of clinical infertility (>1 year of attempts at conception without success) compared to siblings, with 13.0% of survivors taking more than twelve months to achieve pregnancy compared to 8.3% of siblings. In contrast to the CCSS data in this population-based study with three age and deprivation-matched controls for each exposed individual, we have reported the effect by diagnosis and showed an effect across the board for all diagnoses, singularly and in aggregate.

Psychosocial as well as biological factors are likely to have a major impact on the livebirth deficit. Several studies have reported that childhood cancer survivors are less likely to marry and to have children than the general population [18, 27–29], with male survivors of childhood brain tumours being the least likely to marry [27]. In the UK British Childhood Cancer Survivor Study (BCCSS), survivors were less likely to be working than expected (OR (99% CI): 0.89 (0.81–0.98)), and this deficit was greatest for irradiated CNS neoplasm survivors (0.34 (0.28–0.41)). Compared to the general population, survivors were fivefold more likely to be unable to work due to illness/disability; the excess was 15‐fold among CNS neoplasm survivors treated with radiotherapy [30]. It is likely that being less likely to marry/cohabit and hold down employment are contributing factors to the observed reduced chance of a livebirth. The relative contributions of psychosocial factors and biologically reduced fertility to this deficit remain to be more completely studied, but the present and previous analysis point to a complex multimodal impact on when and whether female childhood cancer survivors have a family. Some studies have shown that experiencing cancer as a child or young adult often strengthens the value survivors place on being parents [31]. At the same time, survivors are often unrealistically anxious about the safety of pregnancy and the health risks to their potential offspring. Survivors of childhood cancer who became pregnant in adulthood are not at an increased risk of cancer except when the patient has a cancer-predisposing syndrome [32, 33]. In a population-based Danish study cancer survivors diagnosed before age 35 years were not more likely than their siblings to have children with a chromosomal abnormality [34] and congenital abnormalities are not more common [35]. A study of reproductive intentions of childless adolescent and young adult (AYA) female cancer survivors (diagnosed with cancer between ages 15–35 years) employing California and Texas cancer registries [19] demonstrated that a moderate proportion (22%) of AYA cancer survivors are voluntarily childless, but reproductive intentions were not related to cancer type or cancer treatments. There may be other reasons for voluntary childlessness in cancer survivors, including fear of recurrent disease or genetic risks of passing cancer genes to children. This may explain part of the deficit we have shown across all cancers. In contrast, a Dutch questionnaire cohort study from the Later group [5] of female childhood cancer survivors using siblings and females from the general population as controls found no difference overall in the desire to have children between survivors and controls (86 and 89%, respectively). However, survivors of a CNS tumour were less likely to desire children and survivors without biological children at the time of study were more likely to report that their desire to have children was unfulfilled because of medical reasons (9%), compared to controls (1%).

In our own population-based study [36] of perinatal risks in female cancer survivors treated before the age of 40 years, they were shown to be more likely to give birth before 37 weeks of gestation but did not show an increased risk of low birthweight. A meta-analysis (also including all ages at diagnosis up to 40 years) showed increased risks of both prematurity and low birthweight in the offspring of cancer survivors, mostly related to radiotherapy treatment [35]. In this study of those diagnosed before the age of 18 years there was no significant difference in median birthweight of offspring between survivors versus controls. While there was a small difference in estimated gestation at birth, there was no significant increase in the prevalence of prematurity. While these results are therefore largely reassuring, they also support the need for individualised assessment of obstetric risks and the need for increased antenatal surveillance [23].

### Strengths and limitations

A major strength of this analysis is its size and unbiased population-based data focusing on the chance of livebirth in females under the age of 18 years at diagnosis. For each survivor, there are three age and deprivation-matched controls with adjustment for confounding, whereas other studies have used siblings as controls which may introduce family bias. There are several limitations within our study that require acknowledgement. Having children is by necessity distant in time from diagnosis for most childhood cancer survivors, requiring long-term follow-up data, thus will be incomplete for younger survivors and those diagnosed more recently but the age and diagnostic date matching control for this. More recent birth and death records are currently available for Scotland but only a single additional year (2019), before the extraordinary impacts of the COVID-19 pandemic occurred, might merit inclusion in this study. This would be unlikely to change the overall findings. The birth rate in Scotland in 2021, for example, was among the lowest since records began in 1855. Ascertainment of reproductive outcome and the chance of a livebirth will be complete for those survivors who remain resident in Scotland, but there will be a small number of survivors who have emigrated, and we have no record of their reproductive history. This will also apply to a small percentage of the controls and there is no reason to believe that the survivors or controls are more or less likely to emigrate. This is a population-based linkage study using Scottish cancer and reproductive registries which do not contain treatment exposure data.

In summary in a population-based cohort, we have shown that survivors of cancer diagnosed before their 18th birthday are less likely to have a livebirth when compared to age and deprivation-matched controls. With the availability of new fertility preservation technologies, it becomes increasingly important to offer fertility preservation to girls and young women at the highest risk of infertility [37, 38]. Our study highlights the need for ongoing support for these young women to provide accurate information as to their reproductive function after treatment, and to support their reproductive choices.

## Data Availability

The data that support the findings of this study are available from the eDRIS Team (Public Health Scotland). Restrictions apply to the availability of these data, which were used under licence for this study.
